# Supplementary material for: Different Patterns of HIV-1 Replication in MACROPHAGES is Led by Co-Receptor Usage
Source: Medicina (Kaunas). 2019 Jun 21;55(6):297. doi: 10.3390/medicina55060297 (PMC6630780; doi:10.3390/medicina55060297)
Supplement: Supplementary file 1 [file medicina-55-00297-s001.pdf]

Table S1. Different changes in genes in R5 virus versus X4 virus infected incubated MDM.

	N° Gene	GENE	81A	NL4-3
Apoptosis related genes	1	Fas (TNFRSF6)-associated via death domain	Not activated	Activated
	2	Tumour necrosis factor (TNF superfamily, member 2)	Not activated	Activated
	3	Heat shock transcription factor 2 (Hsf2)	Not activated	Activated
	4	Mitogen-activated protein kinase 10 (c-Jun N-terminal kinase 3)	Not activated	Activated
	5	Matrix metalloproteinase 9 (gelatinase B, 92kD gelatinase, 92kD type IV collagenase)	Not activated	Activated
	6	Matrix metalloproteinase 13 (collagenase 3)	Not activated	Activated
	7	G1 to S phase transition 1 (GSPT1/eRF3)	Not activated	Activated
	8	Signal transducer and activator of transcription 3 (acute-phase response factor) (STAT3)	Not activated	Activated
	9	Fibroblast growth factor 7 (keratinocyte growth factor) KGF	Not activated	Activated
	10	Caspase 7, apoptosis-related cysteine protease	Not activated	Activated
	11	Cytochrome C	Not activated	Activated
	12	Interleukine 16 (lymphocyte chemoattractant factor)	Not activated	Activated
Survival related genes	13	Defender against cell death 1(DAD-1)	Activated	Not activated
	14	Cullin 2 (hCUL2)	Activated	Not activated
